# Evaluation of Tissue and Circulating miR-21 as Potential Biomarker of Response to Chemoradiotherapy in Rectal Cancer

**DOI:** 10.3390/ph13090246

**Published:** 2020-09-14

**Authors:** Susana Ourô, Cláudia Mourato, Marisa P. Ferreira, Diogo Albergaria, André Cardador, Rui E. Castro, Rui Maio, Cecília M. P. Rodrigues

**Affiliations:** 1 Surgical Department, Hospital Beatriz Ângelo, 2674-514 Loures, Portugal; marisa.hferreira@hbeatrizangelo.pt (M.P.F.); diogo.albergaria@hbeatrizangelo.pt (D.A.); rui.maio@hbeatrizangelo.pt (R.M.); 2 NOVA Medical School, Faculdade de Ciências Médicas, 1169-056 Lisboa, Portugal; 3 Research Institute for Medicines (iMed.ULisboa), Faculty of Pharmacy, Universidade de Lisboa, 1649-003 Lisboa, Portugal; cmourato@ff.ulisboa.pt (C.M.); acardador@campus.ul.pt (A.C.); ruieduardocastro@ff.ulisboa.pt (R.E.C.)

**Keywords:** biomarkers, miR-21, chemoradiotherapy, rectal cancer, therapy response, tumor regression grade

## Abstract

Response to chemoradiotherapy (CRT) in patients with locally advanced rectal cancer (RC) is quite variable and it is urgent to find predictive biomarkers of response. We investigated miR-21 as tissue and plasma biomarker of response to CRT in a prospective cohort of RC patients; The expression of miR-21 was analyzed in pre- and post-CRT rectal tissue and plasma in 37 patients with RC. Two groups were defined: Pathological responders (TRG 0, 1 and 2) and non-responders (TRG 3). The association between miR-21, clinical and oncological outcomes was assessed; miR-21 was upregulated in tumor tissue and we found increased odds of overexpression in pre-CRT tumor tissue (OR: 1.63; 95% CI: 0.40–6.63, *p* = 0.498) and pre-CRT plasma (OR: 1.79; 95% CI: 0.45–7.19, *p* = 0.414) of non-responders. The overall recurrence risk increased with miR-21 overexpression in pre-CRT tumor tissue (HR: 2.175, *p* = 0.37); Significantly higher miR-21 expression is observed in tumor tissue comparing with non-neoplastic. Increased odds of non-response is reported in patients expressing higher miR-21, although without statistical significance. This is one of the first studies on circulating miR-21 as a potential biomarker of response to CRT in RC patients.

## 1. Introduction

Rectal cancer (RC) is one of the most prevalent cancers in the world [1] but, despite great progress in treatment options, chemoradiotherapy (CRT) is still ministered in the majority of locally advanced cases [2]. After neoadjuvant treatment, almost 30% of patients exhibit resistance to CRT having no benefit from this therapy [3]. In fact, non-responders are at increased risk of disease progression and toxicity related to CRT. Currently, we cannot predict response and the complications associated with this treatment should not be underestimated. There is an urgent need to identify patients that will not benefit from CRT and thus avoid unnecessary morbidity.

MicroRNAs (miRNAs) are highly conserved non-coding RNAs with a post-transcriptional function of inhibiting mRNA translation. These molecules seem to regulate carcinogenic pathways and the potential role in oncogenesis hypothesized their use as biomarkers in cancer diagnostic and prediction of response to therapy [4]. In fact, miRNAs associated with colorectal cancer (CRC) have been identified in tumor tissue, however, the need for a non-invasive prediction tool prompted their investigation in serum and plasma as circulating markers.

One of the most studied miRNAs is oncomiR-21, demonstrated as a potential diagnostic and prognostic biomarker for CRC, often up-regulated in serum and solid tumors [5,6,7,8,9,10,11,12,13]. In CRC, miR-21 up-regulation has been related to advanced stage, positive lymph nodes, venous invasion, and metastatic behavior [10,11,12,14]. Indeed, miR-21 plays a key role in several biological processes needed for tumorigenesis, including resistance to apoptosis, proliferation, evasion to growth suppressors, replicative immortality, and tumor promoting inflammation [15]. miR-21 oncogenic function is exerted mainly through the suppression of a large number of genes that participate directly or indirectly in the extrinsic or intrinsic apoptosis pathways (PDCD4, PTEN, TPM1, MARCKS, HNRPK, TP63, IL12A, JAG1, BTG2, LRRFIP1, BMPR2, TGFBR2, CDC25A, PELI1, ANKRD46, CDK2AP1, MEF2C, MSH2, MSH6, PPARA, RASGRP1, FASLG, TIMP3, ANP32A, SMARCA4, and THRB). In addition, miR-21 is also a negative regulator of p53 signaling and promotes NF-ĸB, implicated in deregulation of glucose flux and oxidative phosphorylation [15].

However, in rectal cancer (RC) the role of miR-21 as predictor of response to CRT and its association with oncological outcomes has not been fully elucidated. Although one study has demonstrated overexpression of miR-21 in pre-CRT tumor tissue of patients with complete pathological response [16], others have shown that high miR-21 levels associated with worse pathological response, discriminating responders from non-responders [17,18]. Moreover, we have also identified, in a retrospective study, an association between miR-21 expression in pre-CRT rectal tumor tissue and tumor regression grade (TRG), with higher levels correlating with worse pathological response [19]. On the other hand, scarce studies have investigated the potential of circulating miR-21 as a molecular predictor of response in the neoadjuvant therapy setting.

In the present study, using a prospective cohort of patients with RC, we investigated the relation between tissue and plasma miR-21 and evaluated its potential use as a tissue and circulating biomarker of response to CRT. The association between miR-21 and clinical and oncological outcomes was also assessed.

## 2. Results

### 2.1. Patient Clinical Parameters

Clinical and demographic features of all 37 patients are summarized in Table 1.

### 2.2. miR-21 Expression in Responders and Non-Responders

**Figure 1:**
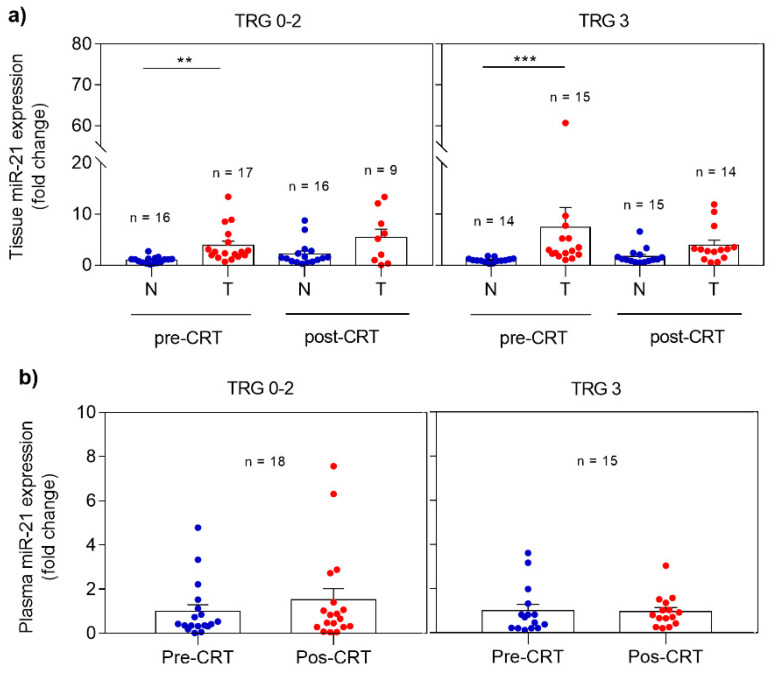
Expression profile of miR-21 in pre- and post-CRT samples in responders (TRG 0-2) and non-responders (TRG 3). (**a**) miR-21 levels in non-neoplastic and tumor tissues; (**b**) miR-21 levels in plasma. Fold changes in tissue and plasma miR-21 expression are calculated from pre-CRT non-neoplastic tissue and pre-CRT plasma expression, respectively. Data are mean ± SEM. N corresponds to non-neoplastic tissue and T to tumor tissue. ** *p* ≤ 0.01, *** *p* ≤ 0.001.

miRNA expression profile was analyzed in non-neoplastic and tumor rectal tissues as well as in plasma, collected before and after CRT. The differences observed when comparing responders (TRG 0-2) and non-responders (TRG 3) are demonstrated in Figure 1. In responders, miR-21 revealed significantly higher expression (*p* = 0.0013) in pre-CRT tumor tissue when compared with non-neoplastic tissue. The same expression profile was observed in post-CRT tissue samples with higher levels of miR-21 in the tumor tissue. However, this profile was also detected in non-responders with overexpression of miR-21 detected in pre-CRT (*p* = 0.0004) and post-CRT tumor tissue when compared with non-neoplastic tissue (Figure 1A).

Regarding miR-21 expression analysis in plasma (Figure 1B), a slight increase with no statistical significance was observed in post-CRT plasma miR-21 expression in responders comparing with pre-CRT samples. Again, no differences were evident before and after treatment in non-responders.

### 2.3. Clinical Parameters and TRG

There was no statistically significant association between clinical parameters and TRG (Table 2). Nevertheless, we observed in our sample a reduced odds of non-response (TGR 3) in women (OR: 0.54; CI: 0.13–2.27; *p* = 0.4), individuals older than 60 years (OR: 0.39; CI: 0.09–1.74; *p* = 0.217), ASA 3 (OR: 0.8; CI: 0.21–3.03; *p* = 0.746), in patients treated with capecitabine based CRT when compared to 5-FU (OR: 0.34; CI: 0.03–4.32; *p* = 0.390) and tumors located in the inferior 1/3 of the rectum (OR: 0.79; CI: 0.21–2.97; *p* = 0.73). On the other hand, the odds of non-response were 6 times higher for cT3 and T4 when compared to cT1 or cT2 (OR: 6.0; CI: 0.64–56.06, *p* = 0.09).

### 2.4. miR-21 Expression and TRG

To study a possible association between miR-21 expression and TRG, we resorted to ROC curve analysis to determine the optimal cut-off that maximized sensitivity, specificity and distinction between responders and non-responders (Table S1). We found increased odds of non-response in patients with higher miR-21 expression (>1.2) in pre-CRT non-neoplastic rectal tissue (OR: 1.2; CI: 0.24–6.06, *p* = 0.828) and in patients with levels higher than 2.61 in pre-CRT tumor tissue (OR: 1.6; CI: 0.40–6.63, *p* = 0.49) (Table 3).

Regarding plasmatic miR-21, there was also an increased odds of TRG 3 in patients with pre-CRT miR-21 expression higher than 0.54 (OR: 1.2; CI: 0.24–6.06, *p* = 0.828) and in patients with post-CRT miR-21 levels >0.84 (OR: 1.09; CI: 0.28–4.33, *p* = 0.9) (Table 3).

Overall, in our sample, patients with higher levels of miR-21 in pre-CRT tissue and plasma had less response to CRT.

### 2.5. Clinical Parameters and miR-21 Expression in Pre-CRT Tumor Tissue and Plasma

In pre-CRT tumor tissue an increased odds of miR-21 overexpression (>2.61 fold change) was observed in patients with cT3-4 (OR: 2.71; 95% CI: 0.44–16.68, *p* = 0.28), TRG 3 (OR: 1.63; 95% CI: 0.40–6.63, *p* = 0.498), local (OR: 1.14; 95% CI: 0.07–20.02, *p* = 0.928) and distant recurrence (OR: 2.73; 95% CI: 0.42–17.65, *p* = 0.289). On the contrary, high miR-21 levels were less likely for subjects older than 60 years (OR: 0.83; 95% CI: 0.19–3.72, *p* = 0.81), obese (OR: 0.38; 95% CI: 0.08–1.69, *p* = 0.21) and ASA 3 (OR: 0.41; 95% CI: 0.09–1.81, *p* = 0.24) (Table 4).

Regarding pre-CRT circulating miR-21, there was an increased probability of miR-21 overexpression (>0.54 fold change) in patients with TRG 3 (OR: 1.79; 95% CI: 0.45–7.19, *p* = 0.414), N+ (OR: 1.75; 95% CI: 0.14–21.44, *p* = 0.663) and distant metastasis (OR: 2.21; 95% CI: 0.07–21.22, *p* = 0.896). However, overexpression was less likely in obese patients (OR: 0.89; 95% CI: 0.22–3.66, *p* = 0.87), cT3 and cT4 (OR: 0.80; 95% CI: 0.14–4.70, *p* = 0.80) and in the presence of distant recurrence (OR: 0.30; 95% CI: 0.07–2.45, *p* = 0.32) (Table 5). Again, overall, patients with miR-21 overexpression in pre-CRT tumor tissue and in blood had less response to CRT.

### 2.6. miR-21 Expression and Oncological Outcomes

**Figure 2:**
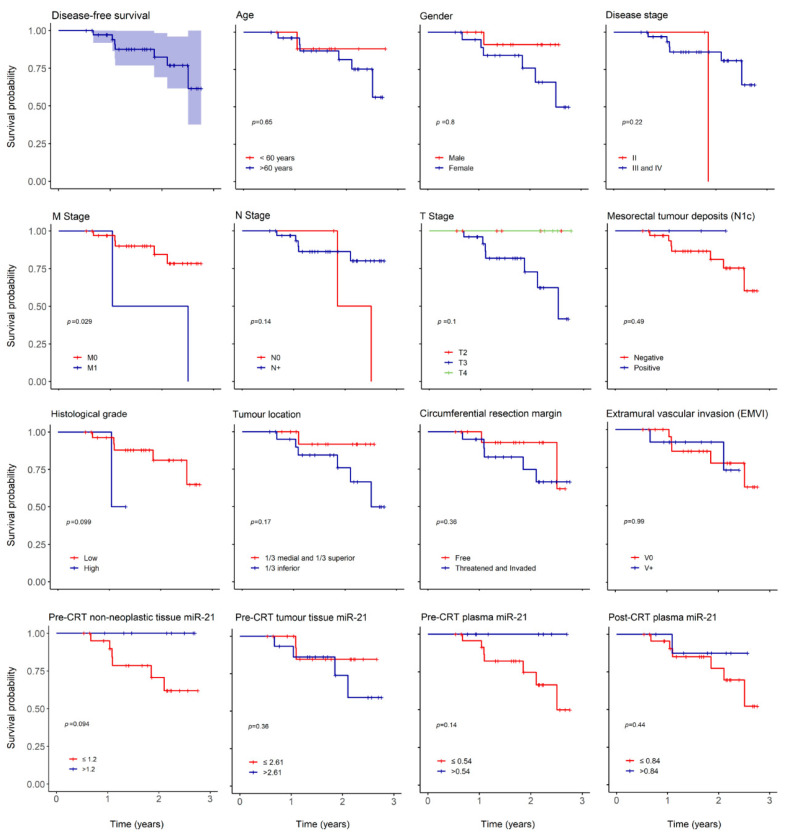
Overall disease-free survival (DFS) and according to clinical and oncological parameters. Kaplan–Meier curves estimating 3-year overall DFS in patients expressing miR-21 and according to age, gender, disease stage, M stage, N stage, T stage, mesorectal tumor deposits (N1c), histological grade, tumor location, circumferential resection margin, extramural vascular invasion (EMVI), pre-CRT non-neoplastic tissue miR-21, pre-CRT tumor tissue miR-21, pre-CRT plasma miR-21 and post-CRT plasma miR-21.

With a median follow up of 603 (196–1007) days, we report 3 (8%) mortality cases, 2 (5%) cases of local recurrence (LR) and 7 (19%) of distant recurrence (DR). The low number of death cases precluded correct estimation of overall survival (OS) but 3 and 5-year predicted disease free survival (DFS) were 67 and 46%, respectively (Figure 2).

The overall recurrence hazard risk (HR) increased in women (HR: 1.218, *p* = 0.797), older patients (HR: 1.64, *p* = 0.65), lower tumor location (HR: 4.03, *p* = 0.19), threatened or invaded circumferential resection margin (CRM) (HR: 2.14, *p* = 0.37) and TRG 3 (HR: 3.95, *p* = 0.11) (Table 6). Overall recurrence HR also augmented in individuals with higher pre-CRT tumor tissue miR-21 expression (HR 2.175, *p* = 0.37) (Table 6).

As expected, there was an impact in 3-year DFS in relation to histological grade (*p* = 0.09) and distant metastasis (*p* = 0.029) (Figure 2) but no influence was noted in age, gender, T or N stage, tumor location, threatened or invaded CRM, N1c or EMVI. There was also a decrease in 3-year DFS in patients with higher pre-CRT tumor miR-21 (*p* = 0.36) and in patients with lower miR-21 in pre-CRT non-neoplastic tissue (*p* = 0.09) and plasma (*p* = 0.14).

We also evaluated the correlation between pre- and post-CRT circulating and tissue miR-21. Results showed, however, very week correlations (Figure S1). There was a positive but frail correlation between pre-CRT plasma and tumor miR-21 with an increase in tissue miR-21 with escalation expression in blood (r = 0.002, *p* = 0.993).

## 3. Discussion

The interest in identifying biomarkers for cancer has led both researchers and clinicians to focus on miRNAs [20]. Some studies have investigated the diagnostic and prognostic value of miR-21 in RC as well as its potential to predict response to CRT [16,17,18]. However, the conclusions obtained from these studies were inconsistent granting the need to further explore the clinical significance of miR-21 as a biomarker in this setting. Generally, findings associate a superior miR-21 expression with a non-or incomplete response. In fact, in a previous retrospective study, our group also identified an association between miR-21 overexpression in pre-CRT rectal tumor tissue and worse pathological response [19]. In that study, this miRNA could differentiate incomplete from complete responders and potentially be used as biomarker to predict TRG. Nevertheless, the evaluation of circulating miR-21 as a non-invasive biomarker of response to CRT in rectal cancer has never been investigated.

The first detection of miRNAs in body fluids occurred when miR-21 was found in the serum of B-cell lymphoma patients [21]. Since then, up-regulated miR-21 levels in plasma have been associated with solid cancers (glioblastoma, breast cancer, and pancreatic cancer) [22] and therefore it was termed oncomiR.

Levels of miRNAs in plasma are remarkably stable, reproducible, consistent among individuals of the same species [23] and cells actively release the majority of circulating miRNAs. The idea of a correlation between circulating and tissue miRNA supports the hypothesis that plasmatic miRNAs can serve as biomarkers of disease or disease response. miRNAs appear to demonstrate the same change in expression, either increased or decreased, in plasma or serum and tumor tissues of patients with various types of cancer [23]. However, only few studies focused on the detection of circulating miRNAs in CRC patients [24,25,26] and this could be attributed to challenges in plasma miRNA extraction and lack of consensus about internal controls for qRT-PCR and normalization.

Clinical significance of circulating miR-21 levels in CRC remains, in fact, not fully understood. Some studies report on seric miR-21 as a discriminative biomarker of colorectal neoplasms from healthy controls [9,27,28,29,30,31,32,33,34,35,36,37] and from benign or premalignant adenoma [33,38]. Circulating miR-21 has also been correlated with tumor size, grade of differentiation, invasion, metastasis [32], recurrence, and survival [6]. The expression of miR-21 has been found significantly increased in preoperative serum from CRC patients who did not received neoadjuvant therapy and this correlated with tumor size, poor survival, and lymph node metastasis [14,37]. Another important issue is that, in reality, very few studies differentiate between colon and rectal cancer patients and these are two different entities with distinct treatment options. In fact, serum miR-21 levels seem to be upregulated in rectum cancer tissue in comparison to colon cancer [39].

In the present work, we aimed to investigate the potential of tissue miR-21 as a biomarker of response to CRT in a prospective cohort of RC patients and validate our previous retrospective results as well as assess circulating miR-21 in this setting. Although we could not demonstrate the efficacy of tissue and plasma miR-21 to differentiate responders (TRG 0-2) from non-responders (TRG 3), we did find an odds increase of non-response in all patients expressing higher miR-21 levels. miR-21 was upregulated in tumor tissue and there was an increased probability of pre-CRT tumor tissue miR-21 overexpression in patients with non-response. In addition, in this study overall recurrence hazard risk increased in patients with less response, threatened or invaded CRM, and higher pre-CRT tumor tissue miR-21 levels. Regarding 3-year DFS analysis, we observed a decrease in survival in patients with higher miR-21 levels in pre-CRT tumor tissue, while overexpression of miR-21 was related to a better survival in pre-CRT non-neoplastic tissue. This is concordant with our hypothesis that when comparing pre-CRT non-neoplastic and tumor tissue we predict response to treatment, where higher miR-21 in pre-CRT tumor tissue in comparison with non-neoplastic tissue is indicative of a worse response to treatment, whereas higher miR-21 in pre-CRT non-neoplastic tissue is associated with better response to CRT. Considering plasma miR-21 analysis, although with no statistical significance, we observed increased odds of pre-CRT circulating miR-21 overexpression in non-responders (TRG 3). Overall, these results are in line with our retrospective study that found a significant association of miR-21 overexpression in pre-CRT rectal cancer tissue with worse response to neoadjuvant therapy [19]. Moreover, pre-CRT plasmatic miR-21 may be also related to less response. To our knowledge, this is one of the first reports in which circulating miR-21 has been investigated as a predictive biomarker of response to neoadjuvant CRT in rectal cancer.

Recently, it was observed that circulating exosomal miR-21 could distinguish chemotherapy resistant from chemosensitive CRC patients [40]. This miRNA was shown to be upregulated in the exossomes of chemoresistant CRC cell lines and in pre-chemotherapy exosomal serum of patients that did not respond to treatment. These results are according to our suggestion that overexpression of pre-CRT circulating miR-21 may be indicative of worse response to CRT in rectal cancer setting, possibly related to the chemotherapy effect. Interestingly, in the present study we also observed a reduced odd of non-response in patients treated with capecitabine based CRT when compared to 5-FU (OR: 0.34; CI: 0.03–4.32; *p* = 0.390). In contrast to 5-FU-based therapies, very limited data is available on miRNA expression and response to CRT with capecitabine. Nevertheless, this outcome lines up with our retrospective study, where 5-FU-treated patients also presented reduced odds of incomplete response (OR: 0.19; 95% CI: 0.03–1.12, *p* = 0.05).

The differences observed between the current work and our previous report, that showed the potential of miR-21 as a discriminative biomarker of response to CRT, are probably due to the limitation in sample size in this prospective study as well as the different TRG based definition of patient groups. Besides, although this group of patients includes uniform sampling and treatment, there is a potential for intratumoral heterogeneity and thus, validation of our results in a larger cohort still needs to be performed.

## 4. Materials and Methods

This was a prospective observational study. Written and signed informed consent for collection and use of biological samples was obtained from all volunteer study participants prior to sample collection. The study protocol conformed to the ethical guidelines of the 1975 Declaration of Helsinki, as reflected in a priori approval by the institutional Human Research Committee and Ethical Committee (Hospital Beatriz Ângelo; 13 March 2017, Project Identification Number 0240). The study was registered in the Portuguese Data Protection Agency.

### 4.1. Patients and Tissue Samples

A total of 37 patients diagnosed with RC (stage I-IV, American Joint Committee on Cancer, AJCC) between April 2017 and June 2019 in the Surgical Department of Hospital Beatriz Ângelo (Loures, Portugal) treated with long course CRT and proctectomy were eligible. Patients had a preoperative staging with pelvic magnetic resonance (MR), thoraco-abdomino-pelvic computed tomography (CT) and endoanal ultrasound when pelvic MR was not clinically possible. Histopathological features were confirmed by pathological analysis and patients were staged according to TNM staging system (8th edition, 2017). Patients with other histological types of rectal malignancy, not submitted to CRT or surgical resection, pregnant or under the age of 18 were excluded.

Two groups of patients were defined: responders (TRG 0, 1, and 2) composed of a total of 21 patients and non-responders (TRG 3) composed of a total of 16 patients.

Fresh frozen tissue samples were collected before and after CRT, during pre-therapeutic colonoscopy and from the protectomy specimen, respectively. Pre-CRT rectal tumor biopsies were gathered from all patients but post-CRT tumor tissues were available only from patients without a pathological complete response. To allow a direct comparison of rectal cancer to matched non-neoplastic rectal mucosa, we collected corresponding adjacent (>1 cm distant) non-tumor tissue both in biopsies and protectomy specimens. Retrieved tumor and non-neoplastic tissue underwent histological confirmation by a pathologist. A fixed amount of tissue (80 μm) was extracted across samples, immediately frozen with CO_2_ prior to storage at −80 °C. In addition, liquid biopsies (plasma) were also collected from 33 patients, before and after CRT, at the time of pre-treatment staging colonoscopy and 24 h after proctectomy. Peripheral blood was collected in vacutainer liquid EDTA 6-mL blood collection tubes and peripheral blood cells and plasma were separated by density gradient separation. Plasma was then stored and frozen at −80 °C until RNA extraction.

### 4.2. Neoadjuvant Treatment

All patients underwent neoadjuvant CRT that consisted of a total dose of 50.4 Gy of pelvic irradiation, 5 times a week, with a daily fraction of 2 Gy using at least a four-field technique. Radiation was delivered with capecitabine (825 mg/m^2^/day) or 5-fluoruocil (5-FU) (1000 mg/m^2^/ day on day 1 to 5 and days 29 to 33). Surgery was performed 10–12 weeks after CRT.

### 4.3. Assessment of Pathological Response

Pathology specimens were graded by Tumor Regression Grade (TRG) according to the College of American Pathologists guidelines (CAP, TNM 7th edition). Two independent pathologists blinded to patient clinical data evaluated TRG categorizing tumors in: TRG 0 or complete response (no viable tumor cells), TRG 1 or moderate score (single cells or little groups of cancer cells), TRG 2 or minimal response (residual cancer outgrown by fibrosis), TRG 3 or poor response (minimal or no tumor killing with extensive residual cancer).

### 4.4. Follow up

Patients had a median of 603 (196–1007) days of follow up with no patients lost.

### 4.5. RNA Isolation from Fresh Frozen Tissues and Serum

Total RNA was extracted using Ribozol^TM^ reagent (VWR International, Radnor, PA, USA) in pre- and post-CRT fresh frozen non-neoplastic and tumor rectal tissues samples according to the manufacturer’s instructions, whereas miRNeasy serum/plasma advanced kit (Qiagen, GmbH, Germany) was used to isolate RNA in pre- and post-CRT plasma samples from a total amount of 200 µL of plasma. In plasmatic RNA isolation, an exogenous control was added to each sample to monitor extraction efficiency and to further normalize miRNA expression data. Thus, 1.6x10^8^ copies/µL of synthetic spike-in control *Caenorhabditis elegans* miR-39 5’-phosphorylated (cel-miR-39-3p_5P) was added according to the miRNeasy kit instructions. RNA extracted from tissue and serum was eluted in 50 µL and 20 µL of RNase-free water, respectively. For a better evaluation of miRNAs quantity in total RNA, the concentration of miRNA was determined using Qubit^TM^ miRNA Assay kit (Invitrogen, ThermoFisher Scientific, Waltham, MA, USA). All RNA samples were stored at −80 °C.

### 4.6. cDNA Synthesis and Real-Time PCR (RT-PCR)

cDNA synthesis was performed using TaqMan^®^ Advanced miRNA cDNA synthesis kit (Applied Biosystems, ThermoFisher Scientific, Waltham, MA, USA) according to the manufacturer’s instructions. Briefly, 2 µL of total RNA (corresponding to 2 ng of RNA extracted from tissue) were extended by a 3′ poly-A tailing reaction and a 5′ adaptor ligation to the mature miRNAs. miRNAs were reverse transcribed into cDNA by reverse transcription using Universal RT primers. In order to improve detection of low-expressing miRNA targets, a pre amplification of the cDNA was performed using the Universal miR-Amp Primers and miR-Amp Master Mix to uniformly increase the amount of cDNA for each target, maintaining the relative differential expression levels. cDNA samples were stored at -20°C. Real-Time PCR was performed on a Quantstudio^TM^ 7 Flex real-time PCR instrument (Applied Biosystems, ThermoFisher Scientific, Waltham, MA, USA) with TaqMan^TM^ Advanced microRNA Assays (Applied Biosystems, ThermoFisher Scientific, Waltham, MA, USA) to assess the expression profile of hsa-miR-21-5p (Assay ID 477975_mir). All reactions were performed in duplicate.

Since a consensual endogenous control for miRNA expression in rectal tissue has still not been determined, normalization was performed with hsa-miR-484 (Assay ID 478308_mir) for tissue miRNA expression analysis. In our previous retrospective study miR-484 was identified as the most stably expressed miRNA with the lowest expression variability when compared with mir-1228-5p, miR-345-5p, miR-103a-3p and the small nuclear (snRNA) U6 and RNU6B, considered endogenous controls for CRC tissues and/or serum. For serum miRNA expression analysis, normalization was performed with cel-mir-39-3p (Assay ID 478293_mir). Expression levels were calculated by the threshold cycle (2^−ΔΔCt^ method), when amplification values were detected. Due to lack of amplification values of miRNAs detected for all tissues, a variable number of samples have been included in each tissue miRNA expression profile. To determine fold change, pre-CRT non-neoplastic tissue and pre-CRT plasma samples were used as controls in tissue and plasma expression analysis, respectively. Fold change values were calculated as the ratio between miR-21 levels in tissue or plasma and the mean of the controls’ values.

### 4.7. Statistical Analysis

miRNA expression was analyzed using the Graph Pad Prism software package, version 7.0 (GraphPad software Inc., San Diego, CA, USA). Normal distribution was determined using the D’Agostino & Pearson omnibus test. Statistical differences between patient groups in plasma expression data were evaluated by two-tailed non-parametric Mann–Whitney *U* test, whereas tissue expression data was analyzed using the one-way analysis of variance (ANOVA) Kruskal–Wallis non-parametric Dunn’s multiple comparison test. Spearman correlation coefficient was used to test the correlation between plasma and tissue miRNA expression levels. Using contingency tables odds ratio (OR) were estimated and the *p*-value associated were obtained resorting to Fisher test. Receiver operating characteristic curve (ROC) were used to calculate optimal cut-offs for miR-21 in pre-CRT normal, tumor tissue and blood determined as the point closest to the top left part of the plot with perfect sensibility and sensitivity. miR-21 was then dichotomized according to these cut-offs. Kaplan–Meier survival curves were compared with Log-rank test and simple Cox proportional hazards models were adjusted to analyze the association of each variable with disease free survival. Overall survival was not possible to determine in this study due to the reduced number of deaths observed (*n*_death_ = 3). Data was analyzed with SPSS (IBM, version 20) and R (version 3.0.2). *p* ≤ 0.05 acknowledged statistical significance. There was professional statistical review performed in this manuscript.

## 5. Conclusions

There is an urgent need for biomarkers of response to CRT. In this study, although the efficacy of tissue and plasma miR-21 to differentiate responders from non-responders could not be demonstrated, the odds of non-response in patients overexpressing miR-21 was increased, however, with no statistical significance. The role of miR-21 as a predictive tool for pathological response in RC patients treated with CRT needs to be established in larger cohorts. Confirmation as such would translate into clinical application through inclusion in algorithms of treatment decision, allowing a better selection of candidates for CRT.

## Figures and Tables

**Table 1:** Patient clinical parameters.

Clinical Parameters		Patients (*n* = 37)
Gender, *n* (%)	Male	25 (68)
	Female	12 (32)
Age, median		62 (42–88)
BMI, median		25 (20–35)
ASA score, *n* (%)	Not discriminated	3 (8)
	I	0 (0)
	II	22 (60)
	III	12 (32)
	IV	0 (0)
Tumor grade	G1/G2	29 (78)
	G3/G4 Not discriminated/determinable	2 (6) 6 (16)
Tumor location (%)	1/3 superior	1 (3)
	1/3 medium	14 (38)
	1/3 inferior	22 (59)
Tumor extension (mm), median		55 (19–90)
Distance to anal verge (mm), median		50 (0–100)
cT	1	0 (0)
	2	7 (19)
	3	25 (68)
	4	5 (13)
cN	0	3 (8)
	+	34 (92)
cM	0	35 (95)
	1	2 (5)
CRM, *n* (%)	Free	17 (46)
	Threatened Invaded	4 (11) 16 (43)
EMVI, *n* (%)	Negative	25 (68)
	Present	12 (32)
c Stage, *n* (%)	I	0 (0)
	II	2 (5)
	III	33 (90)
	IV	2 (5)
CEA (mg/mL), median		1.7 (0.5–96)
CRT	5-FU based	4 (11)
	Capecitabine based	33 (90)
TRG (CAP), *n* (%)	0	9 (24)
	1	7 (19)
	2	5 (14)
	3	16 (43)

BMI: Body Mass Index; ASA: American Society of Anesthesiologists; CRM: circumferential resection margin; EMVI: extramural vascular invasion; CEA: carcinoembrinonary antigen; CRT: chemoradiotherapy; MR: magnetic resonance; TRG: tumor regression grade; CAP: College of American Pathologists.

**Table 2:** Clinical parameters and TRG.

Simple Logistic Regression		OR	95% CI	*p* Value
Continuous Variables				
BMI		1.029	0.2649–3.993	0.968
Age		0.392	0.0887–1.735	0.217
Categorical Variables				
Gender	Female	0.542	0.1291–2.272	0.406
	Male			
Tumor Location	Superior 1/3			
	Medium 1/3			
	Inferior 1/3	0.791	0.2107–2.972	0.732
ASA	1 + 2			
	3	0.800	0.2114–3.028	0.746
CRM MR	Free			
	Threatened, invaded	1.169	0.3162–4.320	0.817
Extramesorectal nodes	Negative			
	Positive	0.542	0.1291–2.272	0.406
cT	T1-2			
	T3-4	6.000	0.6421–56.062	0.090
cN	0			
	+	0.350	0.0289–4.246	0.399
cM	0			
	1	1.333	0.0770–23.085	0.845
Chemotherapy	Capecitabine	0.342	0.0280–4.320	0.390
	5-FU			

Simple logistic regression analysis using TRG as dependent variable (TRG 3) and clinical/ molecular variables as independent variables. OR: odds ratio of non-response (TRG 3); TRG: Tumor regression grade; CI: confidence interval; BMI: body mass index; ASA: American Society of Anesthesiologists; CRM: circumferential resection margin; MR: magnetic resonance.

**Table 3:** miR-21 expression and TRG.

Variables		OR	95% CI	*p* Value
miR-21 pre-CRT non-neoplastic	≤1.2			
	>1.2	1.20	0.237–6.064	0.828
miR-21 pre-CRT tumor	≤2.61			
	>2.61	1.63	0.402–6.625	0.498
miR-21 pre-CRT plasma	≤0.54			
	>0.54	1.20	0.237–6.064	0.828
miR-21 post-CRT plasma	≤0.84			
	>0.84	1.09	0.276–4.330	0.900

Simple logistic regression according to cut-offs determined with ROC curve analysis. OR: odds ratio of non-response (TRG 3); CI: confidence interval.

**Table 4:** Clinical parameters and miR-21 expression in pre-CRT tumor tissue.

Variables		OR	95% CI	*p* Value
Age	<60			
	≥60	0.83	0.19–3.72	0.814
Sex	Male			
	Female	2.1	0.49–8.99	0.322
BMI	Low weight + normal			
	Pre-obesity + obesity	0.38	0.08–1.69	0.206
ASA score	2			
	3	0.41	0.09–1.81	0.242
Stage pre-CRT	I + II			
	III + IV	0.88	0.57–27.24	0.203
cT	T1			
	T3 + 4	2.71	0.44–16.68	0.280
cN	0			
	1	0.87	0.05–15.33	0.928
pTRG	TRG 0 + 1 + 2			
	TRG 3	1.63	0.40–6.63	0.498
Distant recurrence	No			
	Yes	2.73	0.42–17.65	0.289
Local recurrence	No			
	Yes	1.14	0.07–20.02	0.928

Simple logistic regression analysis using miR-21 expression (> 2.61-fold change) as dependent variable and clinical variables as independent variables. OR of miR-21 > 2.61-fold change. OR: odds ratio; CI: confidence interval; ASA: American Society of Anesthesiologists; BMI: body mass index; CRT: chemoradiotherapy; pTRG: pathological tumor regression grade.

**Table 5:** Clinical parameters and miR-21 expression in pre-CRT plasma.

Variables		OR	95% CI	*p* Value
Age	<60			
	≥60	4.14	0.71–24.16	0.106
Sex	Male			
	Female	1.73	0.40–7.46	0.465
BMI	Low weight + normal			
	Pre-obesity + obesity	0.89	0.22–3.66	0.873
ASA score	2			
	3	1.75	0.43–7.17	0.442
Stage pre-CRT	I + II			
	III + IV	0.82	0.05–14.39	0.896
cT	T1 + T2			
	T3 + T4	0.80	0.14–4.70	0.808
cN	N0			
	N1	1.75	0.14–21.44	0.663
cM	M0			
	M1	2.21	0.07–21.22	0.896
pTRG	TRG 0 + 1 + 2			
	TRG 3	1.79	0.45–7.19	0.414
Distant recurrence	No			
	Yes	0.40	0.07–2.45	0.320

Simple logistic regression analysis using miR-21 expression (>0.54-fold change) as dependent variable and clinical variables as independent variables. OR of miR-21 > 0.54. OR: odds ratio; CI: confidence interval; ASA: American Society of Anesthesiologists; BMI: body mass index; CRT: chemoradiotherapy; pTRG: pathological tumor regression grade.

**Table 6:** Clinical parameters, miR-21 levels, and overall recurrence.

Variables		Total	DFS	r Mean	Simple Cox Proportional Hazard Model	
					HR	*p* Value
Tumor location	Superior + medium	15	1	2.53	4.027	0.199
	Inferior	22	6	2.25		
Age	<60	10	1	2.54	1.637	0.651
	≥60	27	6	2.38		
Gender	Male	25	4	2.41	1.218	0.797
	Female	12	3	2.39		
CRM	Free	17	2	2.53	2.135	0.368
	Threatened/invaded	20	5	2.30		
TRG	0–2	21	2	2.57	3.950	0.108
	3	16	5	2.21		
miR-21 pre-CRT tumor	≤2.61	17	2	2.47	2.175	0.37
	>2.61	15	4	2.26		
miR-21 pre-CRT plasma	≤0.54	18	5	2.27	0.464	0.36
	>0.54	15	2	2.45		

Simple Cox Proportional Hazards Model using global recurrence as dependent variable and clinical parameters as independent variables. HR: hazard ratio; CRM: circumferential resection margin; TRG: tumor regression grade; DFS: disease free survival.

## Supplementary Materials

The following are available online at https://www.mdpi.com/1424-8247/13/9/246/s1, Figure S1: Correlation between pre- and post-CRT miR-21 expression in plasma and tumor tissue, Table S1: Predictive value of miR-21 cut-off.

## References

[1] Siegel R.L., Miller K.D., Jemal A. (2019). Cancer Statistics, 2019. CA Cancer J. Clin..

[2] Glynne-Jones R., Wyrwicz L., Tiret E., Brown G., Rödel C., Cervantes A., Arnold D. (2017). Rectal cancer: ESMO clinical practice guidelines for diagnosis, treatment and follow-up. Ann. Oncol..

[3] Dossa F., Chesney T.R., Acuna S.A., Baxter N.N. (2017). A Watch-and-wait approach for locally advanced rectal cancer after a clinical complete response following neoadjuvant chemoradiation: A systematic review and meta-analysis. Lancet Gastroenterol. Hepatol..

[4] To K.K.W., Tong C.W.S., Mingxia W., Cho W.C.S. (2018). MicroRNAs in the prognosis and therapy of colorectal cancer: From bench to bedside. World J. Gastroenterol..

[5] Yu W., Wang Z., Shen L., Qichun W. (2016). Circulating microRNA-21 as a potential diagnostic marker for colorectal cancer: A meta-analysis. Mol. Clin. Oncol..

[6] Menéndez P., Padilla D., Villarejo P., Palomino T., Nieto P., Menéndez J.M., Rodríguez-Montes J.A. (2013). Prognostic implications of serum microrna-21 in colorectal cancer. J. Surg. Oncol..

[7] Shibuya H., Iinuma H., Shimada R., Horiuchi A., Watanabe T. (2011). Clinicopathological and prognostic value of microRNA-21 and microRNA-155 in colorectal cancer. Oncology.

[8] Eslamizadeh S., Heidari M., Agah S., Faghihloo E., Ghazi H., Mirzaei A., Akbari A. (2018). The role of microRNA signature as diagnostic biomarkers in different clinical stages of colorectal cancer. Cell J..

[9] Kanaan Z., Rai S.N., Eichenberger M.R., Roberts H., Keskey B., Pan J., Galandiuk S. (2012). Plasma miR-21: A potential diagnostic marker of colorectal cancer. Ann. Surg..

[10] Kulda V., Pesta M., Topolcan O., Liska V., Treska V., Sutnar A., Rupert K., Ludvikova M., Babuska V., Holubec L. (2010). Relevance of miR-21 and miR-143 expression in tissue samples of colorectal carcinoma and its liver metastases. Cancer Genet. Cytogenet..

[11] Nielsen B.S., Jørgensen S., Fog J.U., Søkilde R., Christensen I.J., Hansen U., Brünner N., Baker A., Møller S., Nielsen H.J. (2011). High levels of microRNA-21 in the stroma of colorectal cancers predict short disease-free survival in stage ii colon cancer patients. Clin. Exp. Metastasis.

[12] Nugent M., Miller N., Kerin M.J. (2011). MicroRNAs in colorectal cancer: Function, dysregulation and potential as novel biomarkers. Eur. J. Surg. Oncol..

[13] de Carvalho T.I., Novais P.C., Lizarte Neto F.S., Sicchieri R.D., Rosa M.S.T., De Carvalho C.A.M., Tirapelli D.P.d.C., Peria F.M., Da Rocha J.J.R., Féres O. (2017). Analysis of gene expression egfr and kras, microRNA-21 and microRNA-203 in patients with colon and rectal cancer and correlation with clinical outcome and prognostic factors. Acta Cir. Bras..

[14] Jin X.H., Lu S., Wang A.F. (2020). Expression and clinical significance of miR-4516 and miR-21-5p in serum of patients with colorectal cancer. BMC Cancer.

[15] White N.M.A., Fatoohi E., Metias M., Jung K., Stephan C., Yousef G.M. (2011). Metastamirs: A stepping stone towards improved cancer management. Nat. Rev. Clin. Oncol..

[16] Eriksen A.H.M., Sørensen F.B., Andersen R.F., Jakobsen A., Hansen T.F. (2017). Association between the expression of microRNAs and the response of patients with locally advanced rectal cancer to preoperative chemoradiotherapy. Oncol. Lett..

[17] Caramés C., Cristóbal I., Moreno V., del Puerto L., Moreno I., Rodriguez M., Marín J.P., Correa A.V., Hernández R., Zenzola V. (2015). MicroRNA-21 predicts response to preoperative chemoradiotherapy in locally advanced rectal cancer. Int. J. Colorectal Dis..

[18] Campayo M., Navarro A., Benítez J.C., Santasusagna S., Ferrer C., Monzó M., Cirera L. (2018). MiR-21, miR-99b and miR-375 combination as predictive response signature for preoperative chemoradiotherapy in rectal cancer. PLoS ONE.

[19] Mourato C., Ourô S., Cardador A., Castro R.E., Albergaria D., Maio R., Rodrigues C.M.P. (2019). miRNAs as molecular predictors of response to chemoradiotherapy in rectal cancer. UEG J..

[20] Zen K., Zhang C.-Y. (2012). Circulating microRNAs: A novel class of biomarkers to diagnose and monitor human cancers. Med. Res. Rev..

[21] Lawrie C.H., Gal S., Dunlop H.M., Pushkaran B., Liggins A.P., Pulford K., Banham A.H., Pezzella F., Boultwood J., Wainscoat J.S. (2008). Detection of elevated levels of tumour-associated micrornas in serum of patients with diffuse large b-cell lymphoma. Br. J. Haematol..

[22] Reid G., Kirschner M.B., van Zandwijk N. (2011). Circulating microRNAs: Association with disease and potential use as biomarkers. Crit. Rev. Oncol./Hematol..

[23] Mitchell P.S., Parkin R.K., Kroh E.M., Fritz B.R., Wyman S.K., Pogosova-Agadjanyan E.L., Peterson A., Noteboom J., O’Briant K.C., Allen A. (2008). Circulating microRNAs as stable blood-based markers for cancer detection. Proc. Natl. Acad. Sci. USA.

[24] Huang Z., Huang D., Ni S., Peng Z., Sheng W., Du X. (2010). Plasma microRNAs are promising novel biomarkers for early detection of colorectal cancer. Int. J. Cancer.

[25] Ng E.K.O., Chong W.W.S., Jin H., Lam E.K.Y., Shin V.Y., Yu J., Poon T.C.W., Ng S.S.M., Sung J.J.Y. (2009). Differential expression of micrornas in plasma of patients with colorectal cancer: A potential marker for colorectal cancer screening. Gut.

[26] Pu X.X., Huang G.L., Guo H.Q., Guo C.C., Li H., Ye S., Ling S., Jiang L., Tian Y., Lin T.Y. (2010). Circulating miR-221 directly amplified from plasma is a potential diagnostic and prognostic marker of colorectal cancer and is correlated with p53 expression. J. Gastroenterol. Hepatol..

[27] Bastaminejad S., Taherikalani M., Ghanbari R., Akbari A., Shabab N., Saidijam M. (2017). Investigation of microRNA-21 expression levels in serum and stool as a potential non-invasive biomarker for diagnosis of colorectal cancer. Iran. Biomed. J..

[28] Peng Q., Zhang X., Min M., Zou L., Shen P., Zhu Y. (2017). The clinical role of microRNA-21 as a promising biomarker in the diagnosis and prognosis of colorectal cancer: A systematic review and meta-analysis. Oncotarget.

[29] Gmerek L., Martyniak K., Horbacka K., Krokowicz P., Scierski W., Golusinski P., Golusinski W., Schneider A., Masternak M.M. (2019). MicroRNA regulation in colorectal cancer tissue and serum. PLoS ONE.

[30] Zhu M., Huang Z., Zhu D., Zhou X., Shan X., Qi L.W., Wu L., Cheng W., Zhu J., Zhang L. (2017). A panel of microRNA signature in serum for colorectal cancer diagnosis. Oncotarget.

[31] Liu H.N., Liu T.T., Wu H., Chen Y.J., Tseng Y.J., Yao C., Weng S.Q., Dong L., Shen X.Z. (2018). Serum microRNA signatures and metabolomics have high diagnostic value in colorectal cancer using two novel methods. Cancer Sci..

[32] Liu Q., Yang W., Luo Y., Hu S., Zhu L. (2018). Correlation between miR-21 and miR-145 and the incidence and prognosis of colorectal cancer. JBUON.

[33] Liu G.H., Zhou Z.G., Chen R., Wang M.J., Zhou B., Li Y., Sun X.F. (2013). Serum miR-21 and miR-92a as biomarkers in the diagnosis and prognosis of colorectal cancer. Tumor Biol..

[34] Shan L., Ji Q., Cheng G., Xia J., Liu D., Wu C., Zhu B., Ding Y. (2015). Diagnostic value of circulating miR-21 for colorectal cancer: A meta-analysis. Cancer Biomark..

[35] Wang B., Zhang Q. (2012). The expression and clinical significance of circulating microRNA-21 in serum of five solid tumors. J. Cancer Res. Clin. Oncol..

[36] Ogata-Kawata H., Izumiya M., Kurioka D., Honma Y., Yamada Y., Furuta K., Gunji T., Ohta H., Okamoto H., Sonoda H. (2014). Circulating exosomal microRNAs as biomarkers of colon cancer. PLoS ONE.

[37] Toiyama Y., Takahashi M., Hur K., Nagasaka T., Tanaka K., Inoue Y., Kusunoki M., Boland C.R., Goel A. (2013). Serum miR-21 as a diagnostic and prognostic biomarker in colorectal cancer. J. Natl. Cancer Inst..

[38] Zhang J., Raju G.S., Chang D.W., Lin S.H., Chen Z., Wu X. (2018). Global and Targeted Circulating microRNA profiling of colorectal adenoma and colorectal cancer. Cancer.

[39] Orosz E., Kiss I., Gyöngyi Z., Varjas T. (2018). Expression of circulating miR-155, miR-21, miR-221, miR-30a, miR-34a and miR-29a: Comparison of colonic and rectal cancer. In Vivo (Brooklyn).

[40] Jin G., Liu Y., Zhang J., Bian Z., Yao S., Fei B., Zhou L., Yin Y., Huang Z. (2019). A panel of serum exosomal microRNAs as predictive markers for chemoresistance in advanced colorectal cancer. Cancer Chemother. Pharmacol..

