# Supplementary material for: Evaluation of Tissue and Circulating miR-21 as Potential Biomarker of Response to Chemoradiotherapy in Rectal Cancer
Source: Pharmaceuticals (Basel). 2020 Sep 14;13(9):246. doi: 10.3390/ph13090246 (PMC7559906; doi:10.3390/ph13090246)
Supplement: Supplementary file 1 [file pharmaceuticals-13-00246-s001.pdf]

SUPPLEMENTARY MATERIALS

Article

Evaluation of Tissue and Circulating miR-21 as Potential Biomarker of Response to Chemoradiotherapy in Rectal Cancer

Susana Ourô [1,2,†,*], Cláudia Mourato [3,†], Marisa P. Ferreira [1], Diogo Albergaria [1], André Cardador [3], Rui E. Castro [3], Rui Maio [1,2] and Cecília M. P. Rodrigues [3,*]

[1] Surgical Department, Hospital Beatriz Ângelo, 2674-514 Loures, Portugal; marisa.hferreira@hbeatrizangelo.pt (M.P.F.); diogo.albergaria@hbeatrizangelo.pt (D.A.); rui.maio@hbeatrizangelo.pt (R.M.)

[2] NOVA Medical School, Faculdade de Ciências Médicas, 1169-056 Lisboa, Portugal

[3] Research Institute for Medicines (iMed.ULisboa), Faculty of Pharmacy, Universidade de Lisboa, 1649-003 Lisboa, Portugal; cmourato@ff.ulisboa.pt (C.M.); acardador@campus.ul.pt (A.C.); ruieduardocastro@ff.ulisboa.pt (R.E.C.)

* Correspondence: smrouro@gmail.com (S.O.); cmprodrigues@ff.ulisboa.pt (C.M.P.R.)

† These authors contributed equally to this work.

Figure

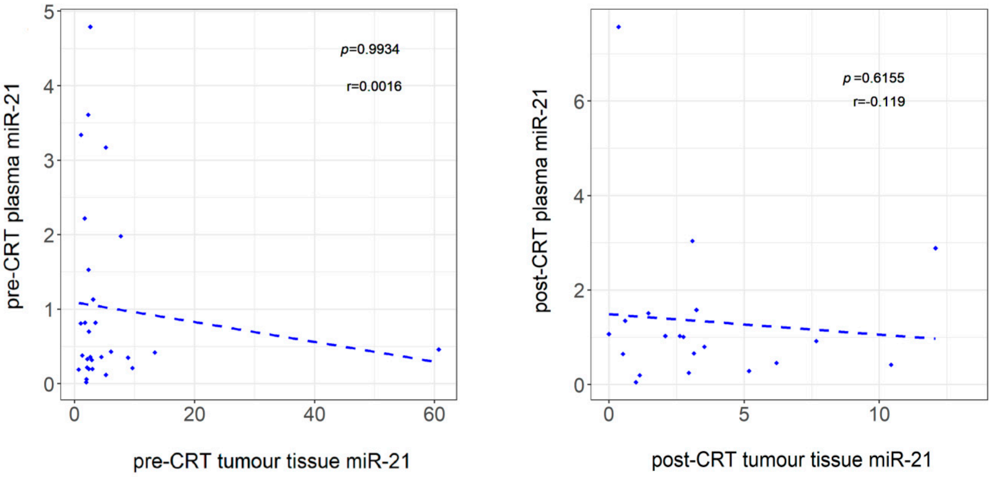

Figure S1. Correlation between pre- and post-CRT miR-21 expression in plasma and tumor tissue.

Table

Table S1. Predictive value of miR-21 cut-off.

miR-21 Cut-Off	Pre-CRT Tumor Tissue 2.61	Pre-CRT Non-Neoplastic Tissue 1.2	Pre-CRT Serum 0.54	Post-CRT Serum 0.84
Sensitivity (%)	53	56	44	50
Specificity (%)	47	57	47	53
PPV (%)	53	60	50	56
NPV (%)	47	53	41	47

Cut-off derived by ROC curve. miR-21 estimated cut-points: 2.61 in pre-CRT tumour tissue; 1.2 in pre-CRT non-neoplastic tissue; 0.54 in pre-CRT serum; 0.84 in post-CRT serum. PPV: positive predictive value; NPV: negative predictive value.
